# Identification of the Key Weather Factors Affecting Overwintering Success of *Apolygus lucorum* Eggs in Dead Host Tree Branches

**DOI:** 10.1371/journal.pone.0094190

**Published:** 2014-04-04

**Authors:** Hongsheng Pan, Bing Liu, Yanhui Lu, Nicolas Desneux

**Affiliations:** 1 State Key Laboratory for Biology of Plant Diseases and Insect Pests, Institute of Plant Protection, Chinese Academy of Agricultural Sciences, Beijing, China; 2 French National Institute for Agricultural Research (INRA), Sophia-Antipolis, France; Ghent University, Belgium

## Abstract

Understanding the effects of weather on insect population dynamics is crucial to simulate and forecast pest outbreaks, which is becoming increasingly important with the effects of climate change. The mirid bug *Apolygus lucorum* is an important pest on cotton, fruit trees and other crops in China, and primarily lays its eggs on dead parts of tree branches in the fall for subsequent overwintering. As such, the eggs that hatch the following spring are most strongly affected by ambient weather factors, rather than by host plant biology. In this study, we investigated the effects of three major weather factors: temperature, relative humidity and rainfall, on the hatching rate of *A. lucorum* eggs overwintering on dead branches of Chinese date tree (*Ziziphus jujuba*). Under laboratory conditions, rainfall (simulated via soaking) was necessary for the hatching of overwintering *A. lucorum* eggs. In the absence of rainfall (unsoaked branches), very few nymphs successfully emerged under any of the tested combinations of temperature and relative humidity. In contrast, following simulated rainfall, the hatching rate of the overwintering eggs increased dramatically. Hatching rate and developmental rate were positively correlated with relative humidity and temperature, respectively. Under field conditions, the abundance of nymphs derived from overwintering eggs was positively correlated with rainfall amount during the spring seasons of 2009–2013, while the same was not true for temperature and relative humidity. Overall, our findings indicate that rainfall is the most important factor affecting the hatching rate of overwintering *A. lucorum* eggs on dead plant parts and nymph population levels during the spring season. It provides the basic information for precisely forecasting the emergence of *A. lucorum* and subsequently timely managing its population in spring, which will make it possible to regional control of this insect pest widely occurring in multiple crops in summer.

## Introduction

Understanding insect population dynamics can be very challenging due to the large number of biotic and abiotic factors that must be taken into account. Of these factors, weather is generally considered to be one of the most important. For instance, temperature, rainfall, relative humidity and other weather-related factors can strongly affect the survival, development, fecundity and behavior of individual insects [1], [2], [3], [4], [5], and the population dynamics of insects [6], [7], [8], [9]. In addition to such direct effects, weather can indirectly impact insect populations by affecting life-history traits of their host plants, competitors, and natural enemies [10], [11], [12].

Global climate change is influencing the geographical distributions and phenology of many insect species [13], [14], [15], [16], some of which are major crop pests. Such changes will inevitably lead to the appearance of new pests in some areas and changes to the biology of well established pests in others [17], [18], [19], [20], Guo et al. [21] found that higher temperatures can lead to earlier egg hatching and eclosion in three Inner Mongolian grasshopper species [*Dasyhippus barbipes* (Fischer de Waldheim), *Oedaleus asiaticus* B. Bienko, and *Chorthippus fallax* (Zubovsky)], which will likely extend their distribution northward as climate change continues. Another good example is the diamondback moth. Indeed, it has been estimated that *Plutella xylostella* (L.) should be able to complete two additional generations for every 2°C of environmental warming [22].

*Apolygus lucorum* (Meyer-Dür) (Hemiptera: Miridae) is the dominant mirid bug species found on many crop species in Northern China, including cotton, Chinese dates, apples, pears, and grapes [23], [24]. Both adults and nymphs feed on plant terminal meristems, young squares and fruits (bolls for cotton), and various other tissues, and results in bushy plants and abscission of squares and fruits (bolls). Eggs are mainly laid inside plant tissues. In each October, *A. lucorum* adults relocate to specific host plants for ovipositing and overwintering eggs [25]. Although *A. lucorum* eggs can successfully overwinter on at least 86 plant species, adults prefer to deposit eggs on fruit trees, such as the Chinese date *Ziziphus jujuba* Mill. and the grape *Vitis vinifera* L. [26]. In April, the overwintering eggs begin to hatch and the newly emerged nymphs feed on tender leaves, buds and flowers. It can seriously damage fruit trees; in particular, the highly mobile nymphs can induce significant cryptic damage which is difficult to be detected, during the initial period of feeding and infestation [27]. Therefore, it is important to determine the factors that affect the hatching rate of overwintering *A. lucorum* eggs in order to develop more precise forecasting techniques.

*A. lucorum* adults generally lay eggs for overwintering in the dead parts of tree branches [26] where the eggs are less likely to be affected by plant growth, which makes weather conditions the most important factor evoking egg hatching. Previous studies showed that temperature and relative humidity strongly affect survival, development, fecundity, and life-history traits of laboratory populations of this insect [28], [29], [30], [31]. In the field, *A. lucorum* as well as other mirid bugs, prefer shady and moist environments [31], and it has been reported that periods of high rainfall result in rapid increases of their populations during the summer [23], [31], [32], [33], [34], [35], [36]. However, the precise effects of factors such as temperature, relative humidity and rainfall on the hatching rate of overwintering *A. lucorum* eggs have not been characterized. The aim of this study was to test the hypothesis that rainfall or other weather factors played a pivotal role in the hatching of *A. lucorum* eggs overwintered in dead host plant (tree) tissues, and then select one or two key weather factors for future forecasting *A. lucorum* population in spring season. We quantified the importance of weather variables on *A. lucorum* hatching by combining with laboratory trials and field surveys.

## Materials and Methods

### Ethics Statement

No specific permits were required for the described field studies.

### Collection of overwintering eggs

Field sampling was carried out in a 1-hectare Chinese date orchard (116.6 °E, 39.5 °N) at the Langfang Experiment Station, Chinese Academy of Agricultural Sciences (CAAS), in Hebei Province, China (116.4 °E, 39.3 °N). Overwintering *A. lucorum* eggs are primarily laid in the summer-pruning wounds of Chinese date trees [25], [35], [37]. During early April, dead branches containing summer-pruning wounds and overwintering eggs were collected from the Chinese date trees and placed in one of four baskets (0.2 m length * 0.2 m width * 0.2 m height) made of bamboo splints. Each basket was affixed with wire to a main branch of a Chinese date tree keeping order to maintain the overwintering eggs under the actual environmental conditions found in the orchard. To eliminate the effects of rain, the baskets were covered with plastic film (0.15 mm thickness, Shandong Jinan Ninglu Economic Plastic Industry Co., Ltd.) prior to each period of rainfall over the course of the experiment; the baskets were then uncovered after the rain had ended.

### Hatching dynamics of overwintering eggs in laboratory conditions

Overwintering *A. lucorum* eggs laid on Chinese date trees primarily hatch between late April and early May in the region of northern China [35], [37]. Therefore, we assessed the effects of various environmental factors on the hatching of overwintering eggs over three time periods: mid April (11^th^ April), late April (21^st^ April), and early May (1^st^ May). At each of these time points, the Chinese date branches containing the overwintering eggs were sampled from the baskets in the orchard, and the numbers of eggs laid on each branch were determined by microscopic examination (SZX16, Olympus, Japan) in the laboratory. To simulate the effects of rain, half of the branches were fully submerged in distilled water for 4 h; the other half of the branches were left dry to act as controls. The treated branches were then placed separately into glass vials (5 cm height ×1.5 cm diameter) and sealed with nylon mesh. Next, the glass vials were placed into environmental growth chambers (Ningbo Jiangnan Instrument Factory, Ningbo, China) adjusted to one of nine combinations of temperature (15, 20, and 25°C) and relative humidity (40, 55, and 70%) according to a completely randomized design with 4 replicates. All chambers were subjected to a photoperiod of 14∶10 (L:D) h, and a total of 100 eggs were tested per replicate. Nymph emergence was recorded daily for 60 d, and newly emerged nymphs were removed from the glass vials. *A. lucorum* nymphs were identified following the keys reported by Lu and Wu [25].

### Hatching dynamics of overwintering eggs in field conditions

Field experiments were conducted at the Chinese date orchard at the CAAS Langfang Experiment Station over five consecutive spring seasons (April 1^st^ through May 31^st^ from 2009 to 2013). No insecticides were applied over the course of the study period. The hatching dynamics of the overwintering *A. lucorum* eggs were monitored every 1–3 days of the observation period. During each sampling, 100–200 branches (30 cm long) with tender leaves were randomly selected and surveyed for the presence of *A. lucorum* nymphs at different instar stages. The emergence of 1^st^-instar nymphs was used to calculate the hatching dynamics of the overwintering eggs.

*Artemisia annua* L., *Artemisia argyi* Lévl. et Vant., *Artemisia lavandulaefolia* DC., and *Humulus scandens* (Lour.) Merr. are the four major host plants of *A. lucorum* adults of last generation prior to egg laying [38], whereas adult females prefer to lay eggs on tree hosts (e.g., Chinese date trees) for overwintering [26]. Therefore, adult population levels on these four fall plants hosts could indirectly influence the abundance of overwintering eggs in nearby tree hosts [26]. To account for this, adult *A. lucorum* population levels on *A. annua*, *A. argyi*, *A. lavandulaefolia*, and *H. scandens* plants near the Chinese date orchard were surveyed using knock-down techniques during early and mid September 2008–2012 [38]. Knock-down techniques basically consisted of pulling parts of the plants over a rectangular 40 cm × 26 cm × 11-cm white-colored pan, after which plant material was struck four times, and the number of dislodged individuals was counted [39].

### Data analysis

A three-way ANOVA test was used to analyze the effects of temperature, relative humidity, rainfall and their interactions, on *A. lucorum* egg hatching. We also analyzed the effects and interactions of temperature, relative humidity, and sampling period on egg hatching rate and developmental duration. One-way ANOVA was used to analyze the effects of temperature or relative humidity on developmental duration. Prior to analysis, egg hatching rates were transformed using the arcsine square root, and developmental duration data were transformed using log_10_(n+1) in order to satisfy the assumption of normality.

Data on temperature, relative humidity and rainfall at the Langfang location between 2009 and 2013 were collected by the Chinese Meteorological Data Sharing Service (http://cdc.cma.gov.cn/). A Simple linear model (y = a + bx) was used for analyzing the relationship between 1^st^-instar *A. lucorum* nymph abundance and (1) all nymphs recorded during that spring (late April to mid May); (2) each weather factor (temperature, relative humidity and rainfall amount) during that spring (mid April to mid May); and (3) adult population levels on different host plants during the previous fall. All the above periods selected for different parameters (inc. *A. lucorum* abundance and weather factors) were determined according to the hatching dynamics of overwintering eggs during the whole study. Prior to analysis, the population numbers of the fall adults and spring nymphs were log_10_(n+1) transformed.

## Results

### Hatching dynamics of overwintering eggs in laboratory conditions

**Figure 1:**
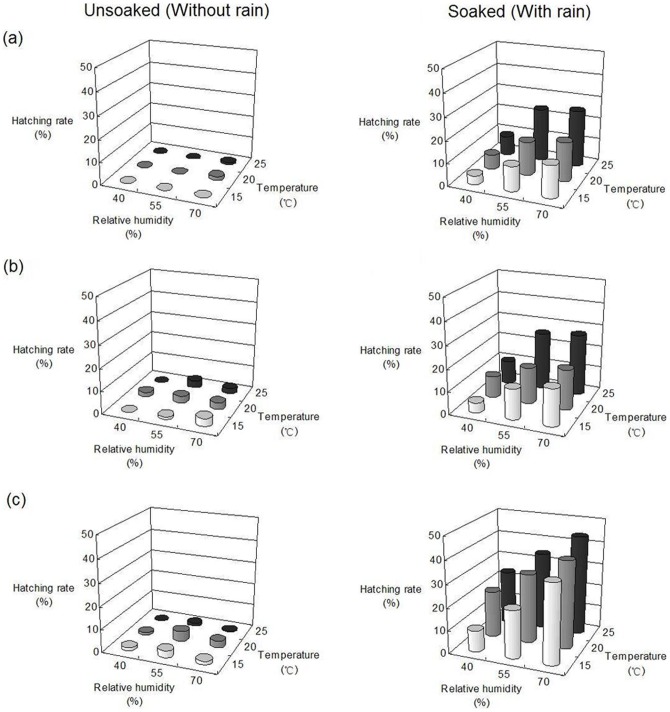
Hatching rates of overwintering *A. lucorum* eggs under different laboratory conditions. (a) Mid April. (b) Late April. (c) Early May.

Temperature, relative humidity, and rainfall, as well as their interaction, affected the hatching rate of overwintering *A. lucorum* eggs (*P*<0.05) (Table 1). Of these factors, rainfall was the most important for egg hatching, since the *F* values for rainfall in the ANOVA tests ranged from 215.07–407.26 during the three study periods (mid April, late April, and early May), while those for temperature and relative humidity ranged from 3.36–18.59 and 12.74–35.59, respectively. Moreover, under conditions of no rainfall, very few nymphs emerged (Figure 1).

**Table 1:** *F*- and *P*-values from the Analyses of Variance tests of the effects of temperature, relative humidity and rainfall (simulated via soaking) on the hatching rate of overwintering *A. lucorum* eggs.

Source of variation	df	*F* value (*P* value)		
		Mid April	Late April	Early May
Temperature	2	18.59 (< 0.0001)	15.67 (< 0.0001)	3.36 (0.0335)
Relative humidity	2	35.39 (< 0.0001)	35.59 (< 0.0001)	12.74 (< 0.0001)
Rainfall	1	407.26 (< 0.0001)	319.49 (< 0.0001)	215.07 (< 0.0001)
Temperature * Relative humidity	4	1.32 (0.2764)	2.21 (0.0810)	0.09 (0.9852)
Temperature * Rainfall	2	16.83 (< 0.0001)	14.24 (< 0.0001)	4.95 (0.0109)
Relative humidity * Rainfall	2	29.42 (< 0.0001)	17.10 (< 0.0001)	10.96 (0.0001)
Temperature * Relative humidity * Rainfall	4	1.28 (0.2886)	0.88 (0.4835)	0.19 (0.9445)

df = degrees of freedom.

**Figure 2:**
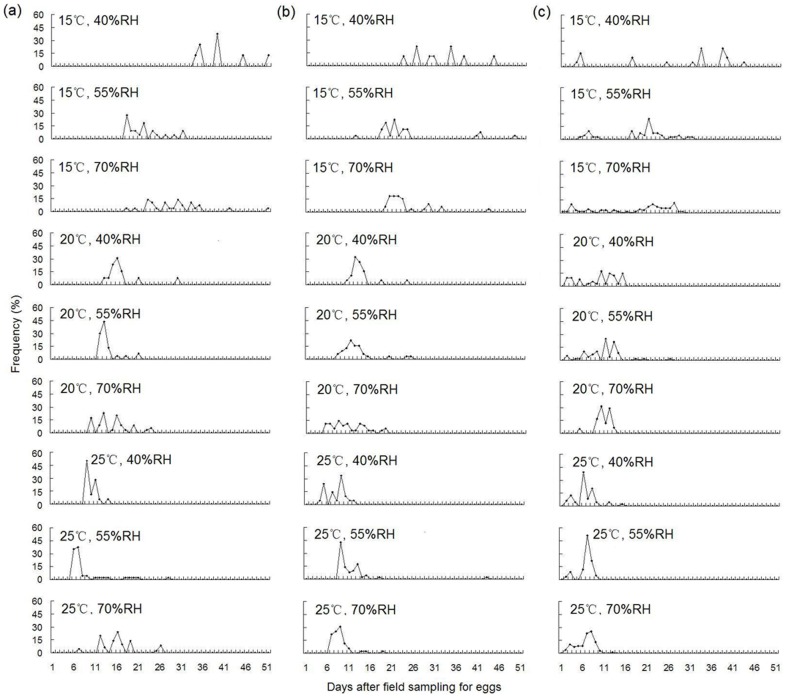
Hatching dynamics of overwintering *A. lucorum* eggs soaked for 4 h under different laboratory conditions. (a) Mid April. (b) Late April. (c) Early May.

We found that egg-hatching rate increased following rainfall, as well as with higher temperatures and relative humidity. Egg-hatching rate also varied according to sampling time. The hatching rate of overwintering eggs sampled in early May was higher than during mid and late April (*P*<0.05), whereas no difference in hatching rate was observed between the latter two periods (*P*>0.05) (Figure 1, Table 2). Furthermore, both temperature and relative humidity significantly affected the developmental duration of the overwintering eggs (*P*<0.05), with temperature playing a stronger role (*F* value = 315.81) over relative humidity (*F* value = 4.70). In other words, high temperatures could hasten the development of overwintering eggs. The developmental duration of the overwintering eggs was different between each sampling period (*P*<0.05) (Table 2, Table 3). For example, the eggs sampled in mid April showed the longest developmental periods as well as the greatest variation in developmental duration between individuals. In contrast, the shortest developmental periods and the least variation in developmental duration between individuals were observed in early May (Table 3, Figure 2).

**Table 2:** *F*- and *P*-values from the Analyses of Variance tests of the effects of temperature, relative humidity and sampling period on the hatching rate and developmental duration of overwintering *A. lucorum* eggs with simulated rainfall (via soaking).

Source of variation	df	*F* value (*P* value)	
		Hatching rate	Developmental duration
Temperature	2	21.25 (< 0.0001)	315.81 (< 0.0001)
Relative humidity	2	45.98 (< 0.0001)	4.70 (0.0119)
Sampling period	2	48.11 (< 0.0001)	61.47 (< 0.0001)
Temperature * Relative humidity	4	0.78 (0.5436)	5.65 (0.0005)
Temperature * Sampling period	4	0.55 (0.7024)	1.67 (0.1667)
Relative humidity * Sampling period	4	2.15 (0.0821)	3.44 (0.0121)
Temperature * Relative humidity * Sampling period	8	0.25 (0.9788)	0.72 (0.6692)

df = degrees of freedom

**Table 3:** The developmental duration of overwintering *A. lucorum* eggs under different combinations of temperature and relative humidity with simulated rainfall (via soaking).

Sampling period	Temperature (°C)	Relative humidity (%)		
		40	55	70
Middle April	15	40.03±3.04 aA	22.49±1.71 aC	29.07±0.60 aB
	20	17.54±1.34 bA	13.57±0.66 bA	15.12±1.03 bA
	25	10.13±0.14 cA	10.55±0.48 cA	12.11±0.85 bA

Late April	15	31.83±2.56 aA	23.47±1.16 aB	23.67±0.62 aB
	20	13.43±1.15 bA	12.26±0.69 bA	10.36±0.70 bA
	25	9.14±0.37 cB	11.61±0.91 bA	8.87±0.18 bB

Early May	15	23.24±5.85 aA	18.56±0.70 aA	17.33±1.13 aA
	20	10.41±2.03 abA	10.62±0.97 bA	10.40±0.19 bA
	25	6.20±0.46 bA	6.62±0.27 cA	6.66±0.38 cA

Note: Different lowercase letters indicate significant differences between the temperature treatments under the same relative humidity within one sampling period; different uppercase letters indicate significant differences between relative humidity treatments at the same temperature within one sampling period (LSD test; *P*<0.05).

### Hatching dynamics of overwintering eggs in field conditions

**Figure 3:**
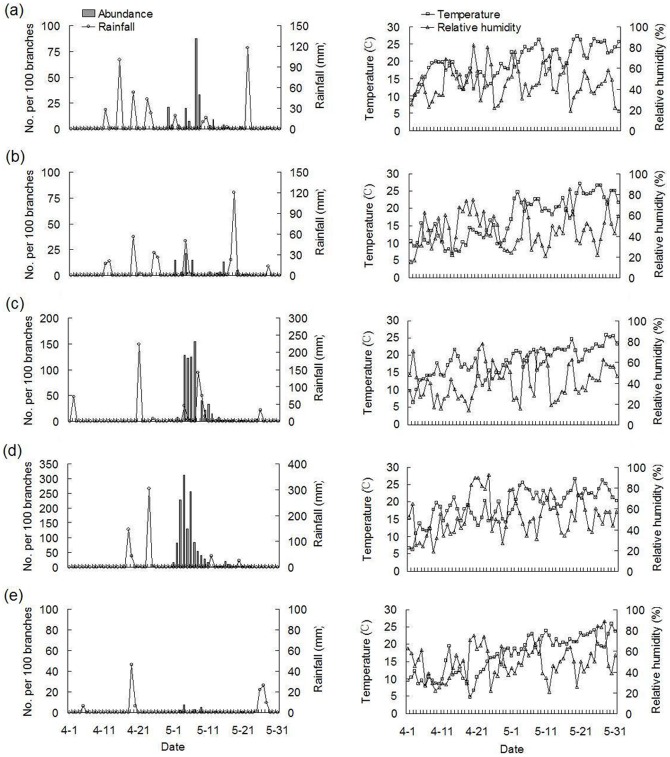
Hatching dynamics of overwintering *A. lucorum* eggs under field conditions during April-May. (a) 2009. (b) 2010. (c) 2011. (d) 2012. (e) 2013.

**Figure 4:**
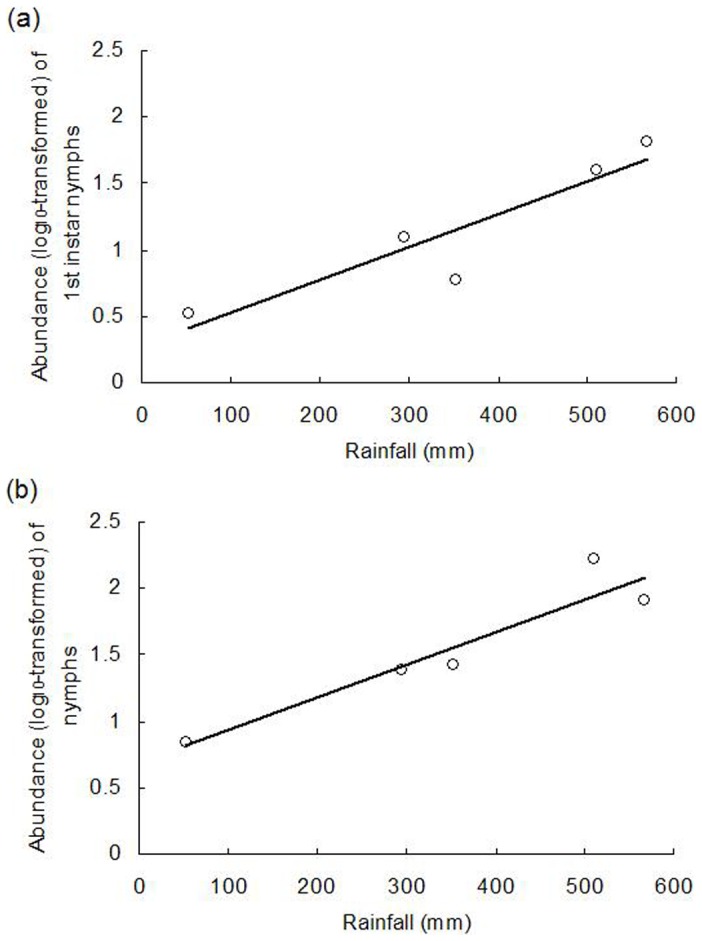
The linear relationships between rainfall and the abundance of *A. lucorum* 1^st^-instar nymphs and nymphs of all stages in a Chinese date orchard during 2009–2013. Linear model: (a) 1^st^-instar nymphs: y = 0.2848 + 0.0025 ×; *F* = 16.82; df = 1,3; *P* = 0.0262. (b) nymphs of all stages: y = 0.6899 + 0.0025 ×; *F* = 24.08; df = 1,3; *P* = 0.0162.

Between 2009 and 2013, a variety of weather conditions (rainfall, temperature, and relative humidity) that were observed have affected the hatching dynamics of overwintering *A. lucorum* eggs and the population levels of newly emerged nymphs (Figure 3). Egg hatching began following a period of rainfall in late April and peaked in early May. The levels of newly emerged nymphs, as well as nymphs of all stages, were positively correlated with rainfall abundance during that spring (*P*<0.05). Neither temperature or relative humidity during the same spring, nor adult abundance on any of the four studied host plants during the previous fall, were correlated with nymph population levels (*P*>0.05) (Table 4, Figure 4).

**Table 4:** *P* values from simple linear regression analyses of the relationships between *A. lucorum* nymph abundance in the spring and (1) weather conditions in the spring and (N) and (2) *A. lucorum* adult abundance the previous fall (N-1).

Period	Parameters	Spring of year (N)	
		Abundance of 1^st^ instar nymphs	Abundance of all nymphs
Fall of year (N-1)	Adult abundance in *Artemisia argyi*	0.4787	0.6042
	Adult abundance in *Artemisia annua*	0.8060	0.8311
	Adult abundance in *Artemisia lavandulaefolia*	0.6806	0.5035
	Adult abundance in *Humulus scandens*	0.3397	0.8075
	Mean adult abundance in these four host plants	0.5011	0.6353
Spring of year (N)	Temperature	0.1404	0.3643
	Relative humidity	0.6782	0.8362
	Rainfall amount	0.0262	0.0162

Note: Fall was defined as the period between early and mid September, and spring as the period between mid April to mid May.

## Discussion

The number of newly emerged *A. lucorum* nymphs in the spring is determined by the initial number of overwintering eggs laid in the fall, the survival rate over the winter (which is dependent on weather conditions), and the hatching rate during the spring season. Very few eggs hatched in the absence of soaking or under conditions of low relative humidity (e.g. 40% RH) following soaking (Figure 1). Consistent with the importance of rainfall and relative humidity for egg hatching and nymph survival [29], the number of 1^st^-instar *A. lucorum* nymphs, as well as nymphs of all stages, was positively correlated with rainfall in the field; no relationships were observed between nymph population levels and the other studied factors. For example, compared with 2011 and 2012, the amount of rainfall was extraordinary low during April-May 2013, which resulted in very few overwintering *A. lucorum* eggs hatching. Niu et al. [35] also reported that the drought directly decreased *A. lucorum* population levels on Chinese dates in spring season. Therefore, we conclude that rainfall is the most important factor for forecasting the population levels of *A. lucorum* during the spring.

In other species it has been noted that specific rainfall characteristics (e.g. intensity, duration and frequency of rainfalls) might exert strong effects on insect population growth and their seasonal dynamics [31], [35], [36]. We described year-to-year fluctuations in the numbers of 1^st^-instar *A. lucorum* nymphs, and it is possible that these fluctuations are due to differences in specific rainfall characteristics. For instance, the intensity, duration, and frequency of rainfall were greater during the 2012 study period than in 2011, which might explain why overwintering eggs hatched earlier in 2012 and why the population density of 1^st^-instar nymphs was markedly higher than in the previous year (Figure 3). Although a similar correlation between nymph population dynamics and specific rainfall characteristics was observed between 2009 and 2010, this relationship requires further study before it can be validated. As changes in rainfall are associated with global climate change [40], it is likely that *A. lucorum* nymph population levels and dynamics will be altered in years to come. The temporal and spatial variation of rainfall distribution also has been recorded in China [41], whereas their potential effects on *A. lucorum* population dynamics need further assessment.

*Apolygus lucorum* overwinters as diapause eggs [42], [43], [44]. Over the course of the study period, which was carried out from mid April to early May, we found that the average length of egg development as well as the variation in development length between individuals greatly decreased. In other words, we found that the rate of diapause termination of the overwintering eggs gradually increased during the study period. It is already known that high temperatures promote diapause termination in overwintering eggs and reduce developmental duration [35], [44]. Consistent with this, we observed that developmental duration became shorter as temperatures increased, given constant relative humidity within the same sampling period (Table 3, Figure 2). Therefore, we conclude that temperature levels should be a useful tool for predicting the developmental status of *A. lucorum* in the field [28], [30].

More than 80 different plant species have been recorded as overwintering hosts for *A. lucorum* [26], some of which are living hosts, whereas others are dead. Eggs that are embedded within live plant tissues share a complex relationship with the host plant, and processes such as egg dormancy and postembryonic development allow egg hatching to coincide with certain phases of host phenology [45]. As a result, eggs generally hatch at a time when food, water and other necessary nutrients are available on the host plant to allow for survival of the young nymphs. In contrast, eggs that hatch on dead plant material can encounter relatively unfavorable conditions for survival and development. However, as *A. lucorum* reportedly feeds upon a variety of common weeds and cultivated plants [25], [33], nymphs that emerge on dead plant material can find necessary resources on surrounding plants.

In this study, we determined that rainfall was the most important weather determinant for the hatching of overwintering *A. lucorum* eggs in the dead parts of tree hosts. In addition, temperature was found to significantly hasten egg development, and relative humidity was found to greatly increase hatching rate. Overall, spring rainfall and temperature were the key factors for predicting population level and emerging period of *A. lucorum* nymphs in this season, respectively. Hence, further forecast the emergence of *A. lucorum* based on weather data will be available for effective management of its spring population, which is the source of *A. lucorum* in different host crops in summer season.
